# Supplementary material for: Transglutaminase 2-expressing macrophages modulate adipose tissue inflammation
Source: Commun Biol. 2025 Jun 4;8:859. doi: 10.1038/s42003-025-08199-1 (PMC12137700; doi:10.1038/s42003-025-08199-1)
Supplement: Supplementary file 2 — Supplementary information [file 42003_2025_8199_MOESM2_ESM.pdf]

Supplementary Information for:

Transglutaminase 2-expressing macrophages modulate adipose tissue inflammation.

Diana M. Elizondo, Tushar P. Patel, Benjamin T. Cole, Eliza A. Jansujwicz, Jocelyn Chen, Melanie C. Hollis, Apratim Mitra, Jack A. Yanovski

Supplementary Figure S1

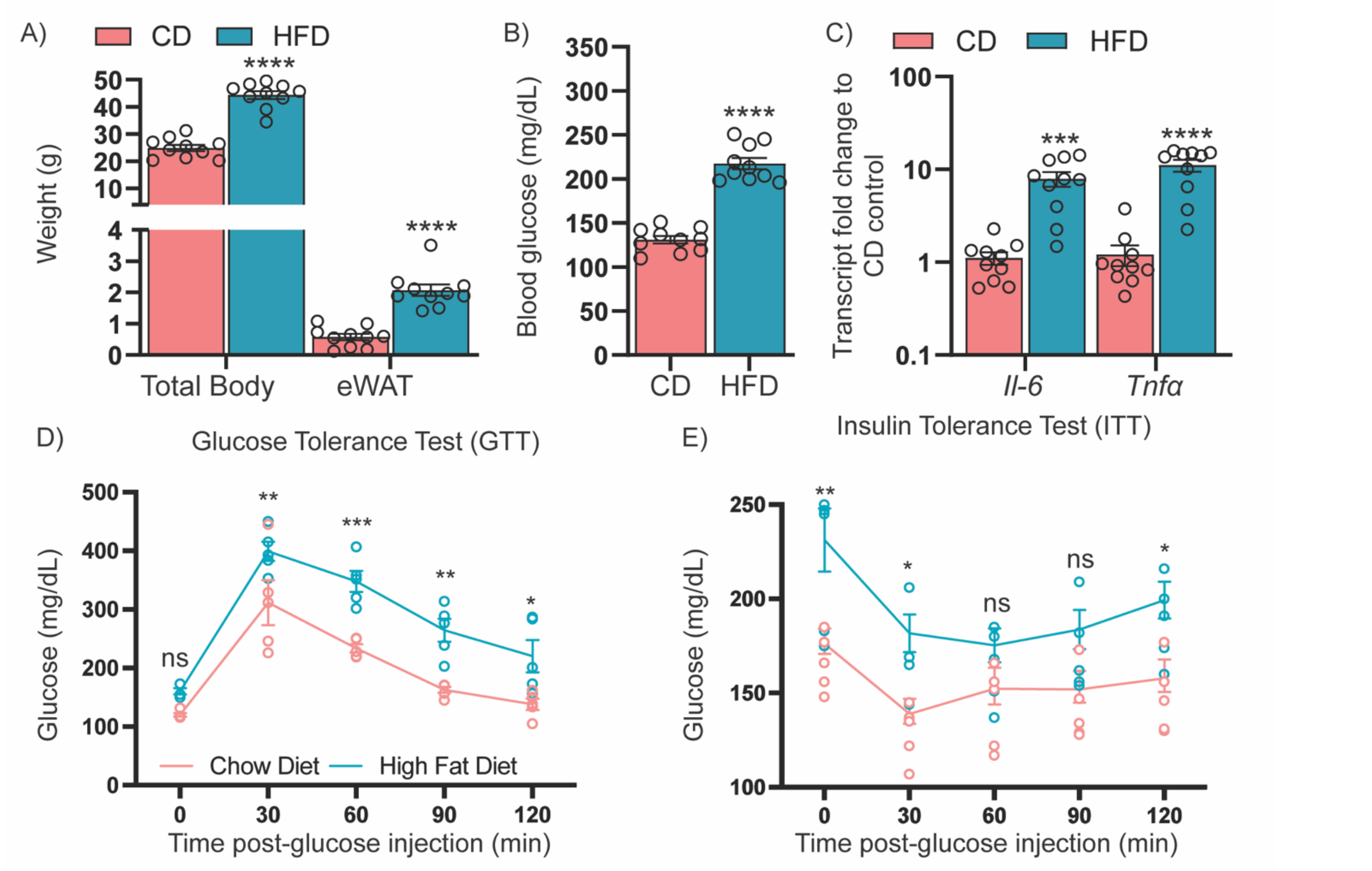

Supplemental Figure S1: **High fat diet (HFD)-induced obese mouse model characterization**. C57BL/6J male mice were placed on chow diet (CD) or 60% high fat diet (HFD) at 6 weeks of age and remained on diet treatment until 16 weeks of age (n=10/diet). **A**) CD vs. HFD total body and epididymal white adipose tissue (eWAT) weight and **B**) non-fasting blood glucose concentrations post- diet treatments. **C**) qPCR analysis of inflammatory markers' mRNA expression in eWAT from CD vs. HFD mice. **D**) GTT in CD and HFD mice. **E**) ITT in CD and HFD mice. Data are shown as SEM of five or ten mice per control or treated group and are representative of two to three independent experiments. For graphs A-C, statistical significance between group was determined by student's unpaired t-test, or non-parametric Mann-Whitney test, depending on data normality. GTT and ITT data were analyzed by Repeated Measures ANOVA followed by t-tests between timepoints. ns=P>0.05, *= P<0.05, ** = P<0.01, ***P<0.001.

Supplementary Figure S2

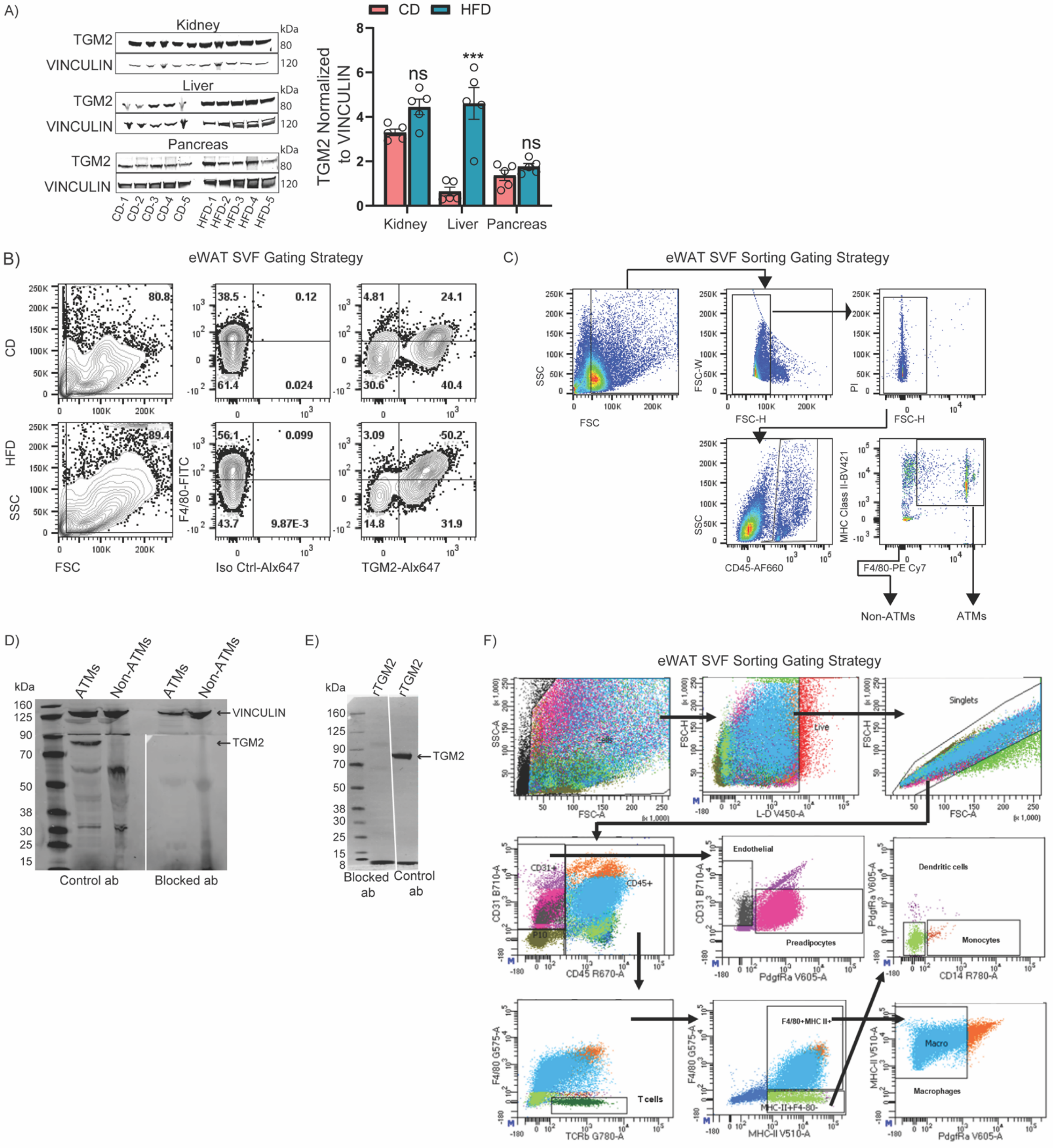

Supplementary Figure S2: TGM2 expression distribution in CD vs. HFD tissues and adipose tissue cells. **A**) TGM2 expression in other metabolically involved tissues (kidney, liver and pancreas) from CD vs. HFD mice (n=4-5/group). **B**) Flow cytometric gating strategy evaluating abundance of TGM2 expressing ATMs in CD vs. HFD treated mice. **C**) Gating strategy for sorting of adipose tissue macrophages from HFD induced obese mice epididymal white adipose tissue (eWAT). Isolated SVF was stained extracellularly for CD45, MHC Class II, F4/80, CD11b and CD11c markers and utilized for sorting in BD FACS Fusion sorter. CD45+ MHC Class II+ F4/80+ cells were labeled as ATMs, while CD45+ MHC Class II- F4/80- cells were labeled as Non-ATMs. Both cell subsets were immediately collected in PBS and re-suspended in lysis buffer for protein lysate preparation and further **D**) evaluation of TGM2 protein expression in sorted ATMs and Non-

ATMs employing control anti-TGM2 antibody and blocked anti-TGM2 antibody for specificity testing (n=5 mice's eWAT SVF sorted cell pellets pulled together). **E**) Recombinant TGM2 (rTGM2) protein was assayed against TGM2 primary antibody blocked by TGM2 native protein for 2h at room temperature (Lane 1), and control TGM2 antibody at working concentration (Lane 2) to test for anti-TGM2 antibody specificity. **F**) Gating strategy for sorting of preadipocytes, macrophages and monocytes from eWAT-derived SVF from CD vs. HFD mice. Data are shown as individual results with bar graph for mean ± SEM from ten mice. Statistical significance was determined by student's unpaired t-test, or non-parametric Mann-Whitney test, depending on data normality.

Supplementary Figure S3

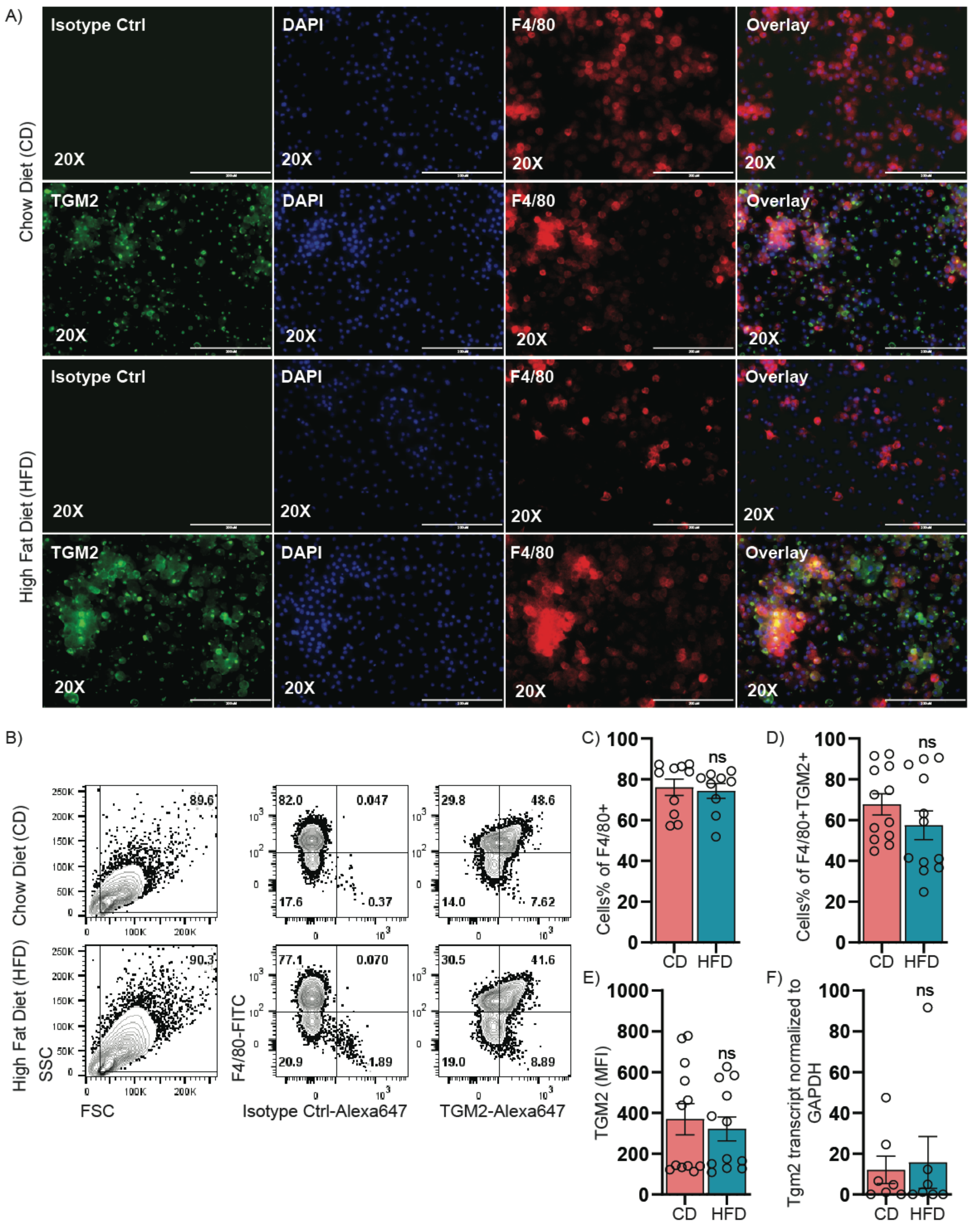

Supplementary Figure S3: TGM2 expression in Bone Marrow Derived Macrophages (BMM) from diet-induced obese mice. C57BL/6J mice were placed on Chow diet (CD) or 60% high fat diet (HFD) for ten weeks. Respective long bones were dissected to isolate bone marrow and used for preparation of bone marrow-derived macrophages (BMMs) *ex vivo*. **A**) Fluorescence microscopy of BMMs stained for TGM2 (or

isotype control), F4/80, and DAPI. **B**) Flow cytometric gating strategy of BMM stained with F4/80 and TGM2-targeting antibodies. Flow cytometric analysis graphing **C**) F4/80+ and **D**) F4/80+TGM2+ BMM cells% and respective measured **E**) TGM2 MFI. **F**) qPCR assessment of TGM2 in differentiated BMMs. Data are shown as individual results with means denoted by bar graphs ± SEM from twelve mice per control or treated group collected in four independent experiments. For all graphs, statistical significance was determined by student's unpaired t-test, or non-parametric Mann-Whitney test, depending on data normality.

Supplementary Figure S4

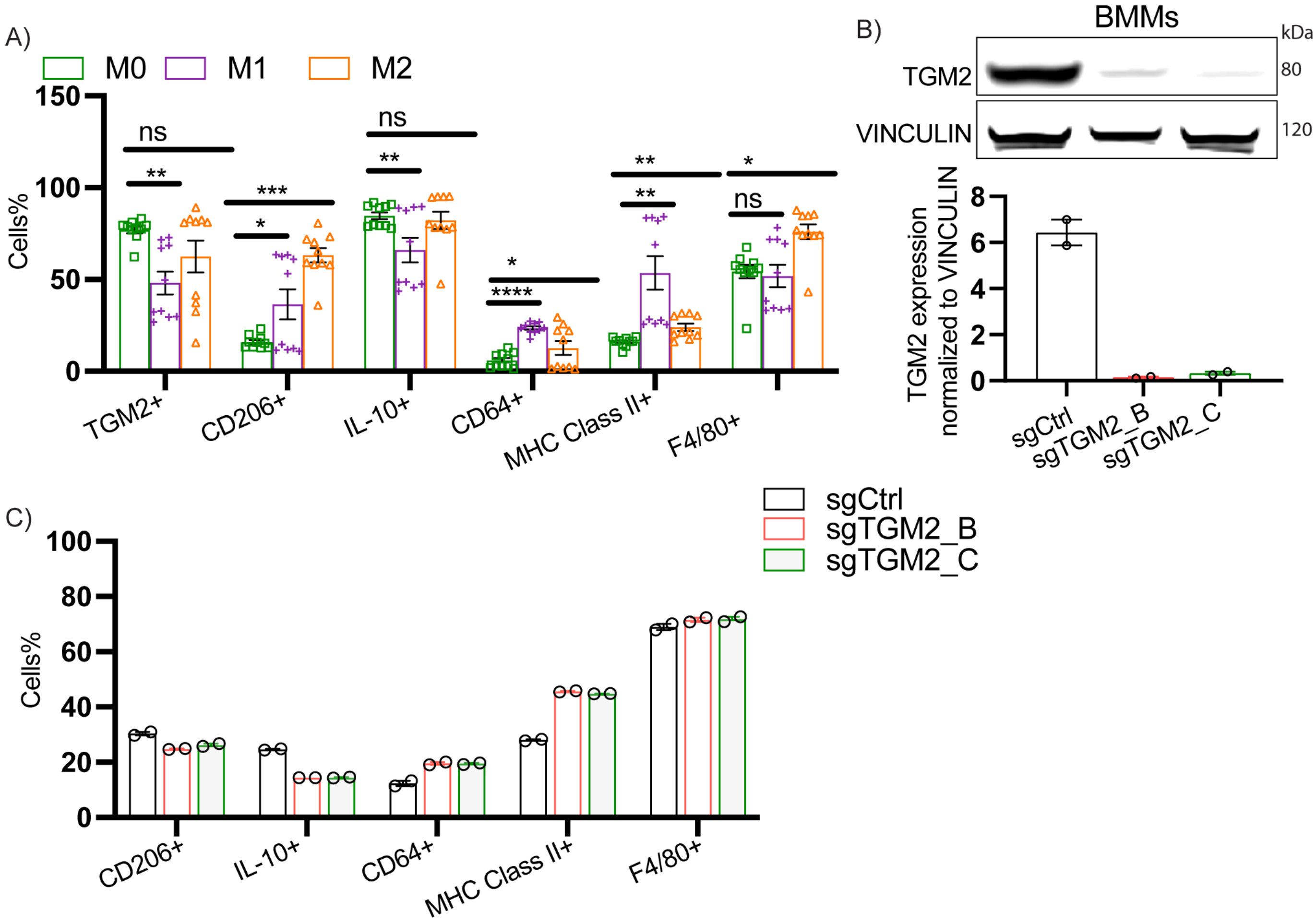

Supplementary Figure S4: Bone marrow macrophage *Tgm2* CRISPR silencing effects. Bone marrow was isolated from C57BL/6J mice and cultured in the presence of M-CSF to induce BMMs differentiation for 4 days. BMMs were then transfected with CRISPR targeting *Tgm2* and evaluated 72h post-transfection via flow cytometric analysis. **A**) Analysis of M0/M1/M2 polarizing effects on macrophage marker expression in sgCtrl BMMs. **B**) Western blot analysis of *Tgm2* silencing efficiency from additional sgTGM2_B and sgTGM2_C sgRNA sequences and **C**) Flow cytometric assessment of M1/M2 marker expression in M0, sgCtrl vs. sgTGM2_B or sgTGM2_C BMMs. Data are shown as bar graphs with mean ± SEM from 4-10 mice per control or treated group collected in two to three independent experiments. For all graphs, statistical significance was determined by student’s unpaired t-test, or non-parametric Mann-Whitney test, depending on data normality.

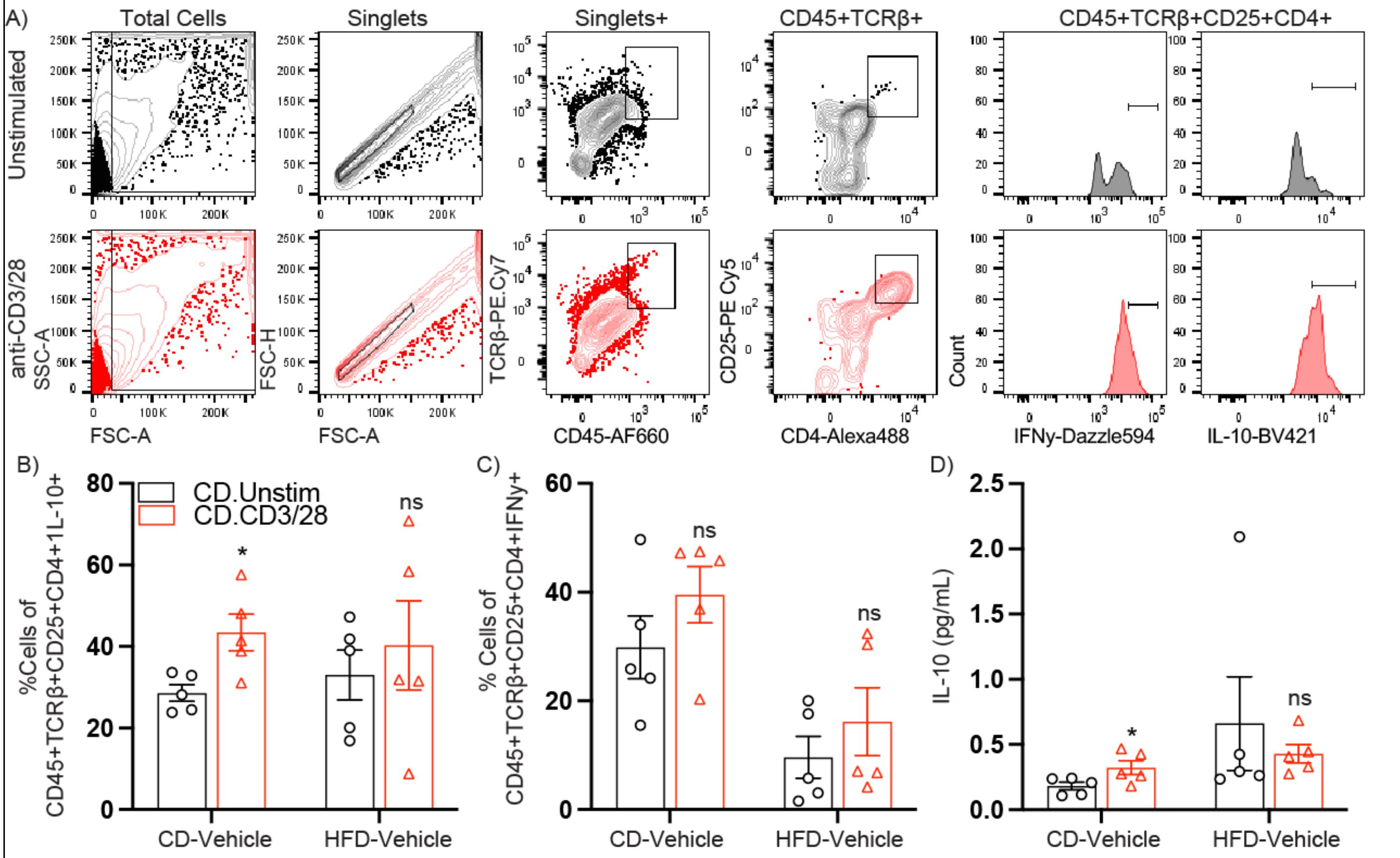

Supplementary Figure S5: In vitro T cell activation efficiency in CD and HFD-derived SVF cells. eWAT SVF cells were harvested from CD or HFD C57BL/6J mice and processed for single cell suspension, followed by ACK red blood lysis treatment. Cells were then plated in a 96-U-bottom well plate in the presence of anti-CD3/CD28 stimuli (or unstimulated control). Harvested cells were assessed with flow cytometry analysis gating on A) CD45+TCRb+CD25+CD4+ T cell population for B) IL-10 and C) IFNg expression. D) ELISA of IL-10 secretion analysis in harvested supernatant. Data are shown as individual results and bar graphs showing mean ± SEM from 5 mice per control or treated group collected in two independent experiments. For all graphs, statistical significance was determined by student's unpaired t-test, or non-parametric Mann-Whitney test, depending on data normality.

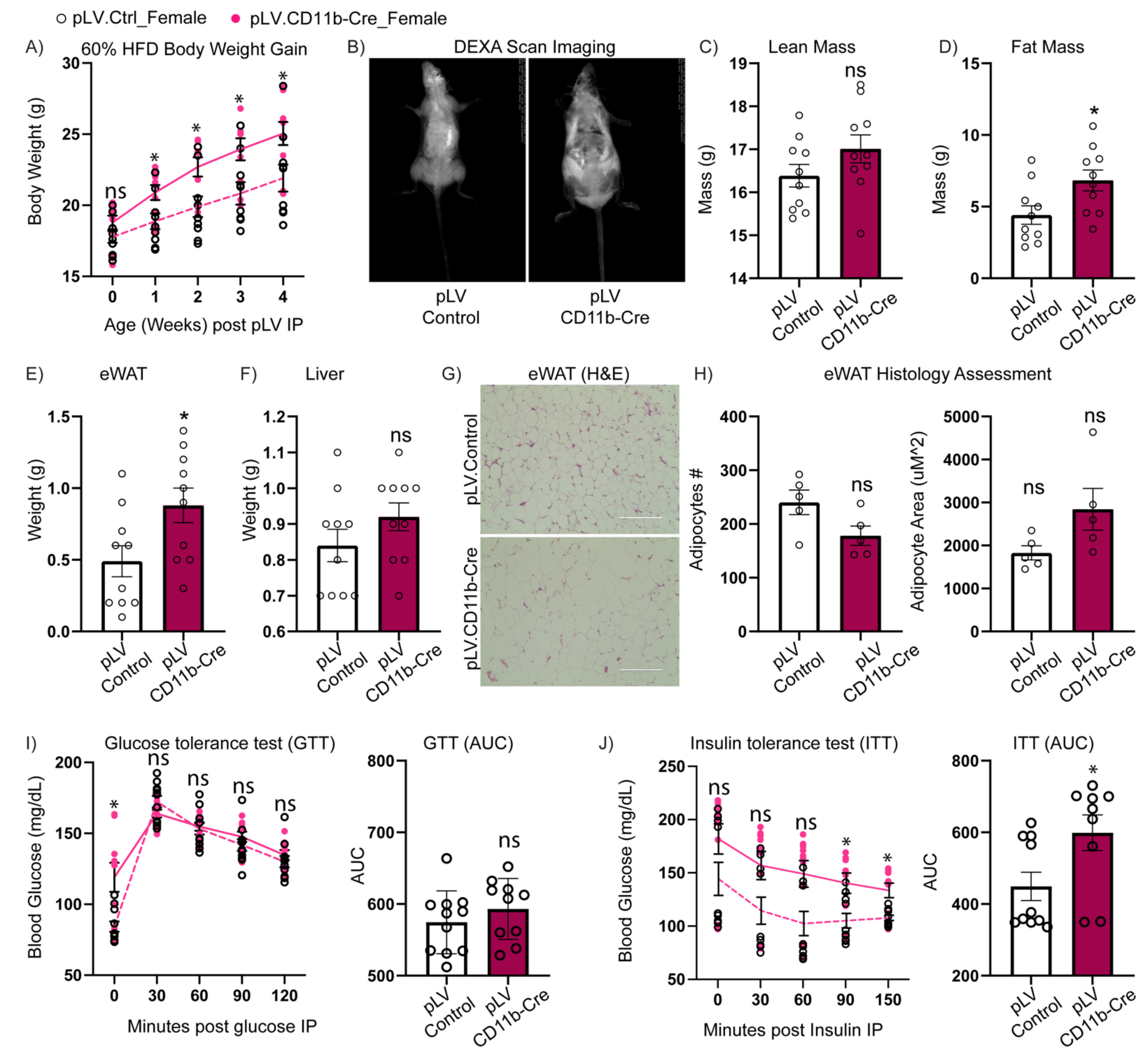

Supplementary Figure S6: ***In* vivo silencing of *Tgm2* in myeloid cells effects in female mouse metabolic health**. Tgm2 female floxed homozygous mice injected with pLV.Control or pLV.CD11b-Cre lentiviruses at 5weeks of age were then placed on a 60%HFD a week later. Mice were subsequently monitored for metabolic changes. A) Weekly weight monitoring. B) DEXA scanning evaluating changes in C) lean and D) fat mass. E) gross eWAT and F) liver weights post-study termination. G) eWAT H&E histology and H) respective histology analysis evaluating number of adipocytes and adipocyte area in pLV.Control vs. pLV.CD11b-cre treated mice (n=5/group). Whole animal metabolic health assessment by I) GTT and J) ITT evaluations (n=10/group). Data are shown as bar graphs with SEM of five mice per control or treated group and are representative of two independent experiments. For body weight, GTT (group: P=0.1323, time: P=<0.0001 and group x time interaction: P=<0.0001) and ITT (group: P=0.0194, time: P=<0.0001 and group x time interaction: P=<0.0001) statistical significance was determined by repeated measure Two-way ANOVA post-hoc. For bar graphs, statistical significance was determined by student's unpaired t-test, or non-parametric Mann-Whitney test, depending on data normality. ns=P>0.05, *= P<0.05, ** = P<0.01, ***P<0.001.

Supplementary Table 1

Materials and Reagents	Company	Identifier
**qPCR probes**		
FAM-labeled *Il6*	Thermo Fisher	Mm00446190_m1
FAM-labeled *Tnfa*	Thermo Fisher	Mm00443258_m1
FAM-labeled *Tgm2*	Thermo Fisher	Mm05905055_s1
FAM-labeled *Tgm4*	Thermo Fisher	Mm01341293_m1
FAM-labeled *Ifng*	Thermo Fisher	Mm01168134_m1
Mrc1	IDT	Mm.PT.5842560062
il-10	IDT	Mm.PT.58.13531087
il-6	IDT	Mm.PT.58.10005566
ifng	IDT	Mm.PT.58.41769240
VIC-labeled *Gapdh*	Thermo Fisher	Mm99999915_g1
VIC-labeled *Actb*	Thermo Fisher	Mm02619580_g1
**Antibodies and recombinant proteins**		
Mouse IgG1 kappa Isotype Control	eBioscience	Cat#14-4714-82
Purified Rat Anti-Mouse CD16/CD32 (Mouse BD Fc Block™)	BD Biosciences	Cat#553141
Goat anti-Mouse, Alexa Fluor 647	Thermo Fisher	Cat#A-21238
Donkey anti-rabbit, Alexa Fluor 647	Biolegend	Cat#406414
Goat anti-Mouse, Alexa Fluor 488	Thermo Fisher	Cat#A-11001
Anti-Mouse, APC.Cy7	Biolegend	Cat#405715
Goat anti-Mouse IgG, DyLight™ 350	ThermoFisher	Cat#62271
Goat anti-Mouse IRDye 680RD	LI-COR	926-68070
TGM2	Thermo Fisher	Cat#MA5-12739
TGM2	Abcam	Cat#ab109121
B-ACTIN	Abcam	Cat#ab8229
VINCULIN	Abcam	Cat#ab129002
ADRP/Perilipin 2	Thermo Fisher	Cat# CL59415294100UL
F4/80-Alexa 594	Biolegend	Cat#123140
F4/80-PE	Biolegend	Cat#111603
CD206 – BV605	Biolegend	Cat#C068C2
CD206- Alexa488	Biolegend	Cat# 141709
CD14-APCCy7	Biolegend	Cat#123317
MHC Class II - BV510	Biolegend	Cat#107635
MHC Class II – Brilliant Violet 650	Biolegend	Cat#107641
IL-10 – BV421	Biolegend	Cat#505022
CD4-Alexa488	Biolegend	Cat#100425
IL-10 – APC Cy.7	Biolegend	Cat#5050335
MHC Class II-PerCp-eFluor710	Thermo Fisher	Cat#46-5321-82
F4/80-PE Cy.7	Biolegend	Cat#123113
CD11c – Alexa594	Biolegend	Cat#117346
CD11c-Brilliant Violet 421	Biolegend	Cat#117329
CD11b-PerCP/Cyanine5.5	Biolegend	Cat#101227
CD206 – Brilliant Violet 421	Biolegend	Cat#141717

CD64-FITC	Thermo Fisher	Cat# MA5-46784
CD4-Alexa488	Thermo Fisher	Cat# 53-0041-82
CD31-PerCPCy5.5	Biolegend	Cat#160206
CD140a-BV605	Biolegend	Cat#135916
TCRb – PE Cy.7	Biolegend	Cat#109222
TCRb-PercP Cy5.5	Biolegend	Cat#109227
CD25 – PE Cy.5	Biolegend	Cat#102010
IFNg – Dazzle 594	Biolegend	Cat#505846
CD45 – Alexa Fluor 660	Thermo Fisher	Cat# 606-0451-82
Live/Dead- V450	Thermo Fisher	Cat#65-0863-14
M-CSF	PeproTech	Cat#315-02
recombinant TGM2 (rTGM2)	R&D Systems	Cat#5418-TG-010
Recombinant Murine IFN-γ	Peprotech	Cat# 315-05
Recombinant Murine IL-4	Peprotech	Cat# 214-14
**Lentivirus**		
p LV[Exp]-EGFP/Puro-EF1A>mCherry	Vector Builder	Vector ID: VB010000-9298rtf
pLV[Exp]-EGFP-CD11b>Cre	Vector Builder	Vector ID: VB230425-1457ejv
**Plastics, Chemicals and Assays**		
Polycarbonate Cell Culture Inserts, 0.4um pore size in 6-well plate	Thermo Fisher	Cat#07-200-165
RNeasy Lipid Tissue Mini Kit	Qiagen	Cat#74804
cDNA reverse transcription kit	Thermo Fisher	Cat#4374966
TaqMan Fast Master Mix	Thermo Fisher	Cat#4444556
SYBR™ Green Master Mix	Thermo Fisher	Cat#A25743
Transglutaminase activity assay	Abcam	Cat#ab204700
protease inhibitor cocktail with EDTA	IBI Scientific	Cat#IBI01150
NuPAGE™ 4 to 12%, Bis-Tris, 1.0 mm, Midi Protein Gels	Thermo Fisher	Cat#WG1401BOX
iBlot™ 2 Transfer Stacks, nitrocellulose, regular size	Thermo Fisher	Cat#IB23001
DAPI	Thermo Fisher	Cat#AMEP4650
Propidium Iodide	Thermo Fisher	Cat#P1304MP
Cas9 nuclease	Thermo Fisher	Cat#A36496
sgTGM2	Thermo Fisher	Cat#A35533; Assay ID: CRISPR474452_SGM
sgTGM2_B	Thermo Fisher	Cat #A35533; Assay ID: CRISPR474465_SGM
sgTGM2_C	Thermo Fisher	Cat #A35533; CRISPR474474_SGM
sgNegative control	Thermo Fisher	Cat# A35526
Cas9 Plus Reagent	Thermo Fisher	Cat# CMAX00001
Mouse Transglutaminase 2/TGM2 ELISA	RayBiotech	Cat#ELM-TGM2-1
LEGENDplex™ Mouse IFN-γ Capture Bead A6, 13X	Biolegend	Cat#740153
LEGENDplex™ Mouse TNF-α Capture Bead A7, 13X	Biolegend	Cat#740154
LEGENDplex™ Mouse IL-6 Capture Bead B4, 13X	Biolegend	Cat#740159

LEGENDplex™ Mouse Inflammation Panel Standard	Biolegend	Cat#740371
LEGENDplex™ Mouse Inflammation Panel Detection Antibodies	Biolegend	Cat#740165
LEGENDplex™ Buffer Set B	Biolegend	Cat#740373
LEGEND MAX™ Mouse IL-10 ELISA Kit	Biolegend	
e Bioscience™ Lipopolysaccharide (LPS) Solution (500X)	Thermo Fisher	Cat# 00-4976-93
αCD3/CD28 Dynabeads	Invitrogen	Cat#11456D
Brefeldin A solution	Thermo Fisher	Cat#00-4506-51

Uncropped/unedited blot/gel images

Figure 1C

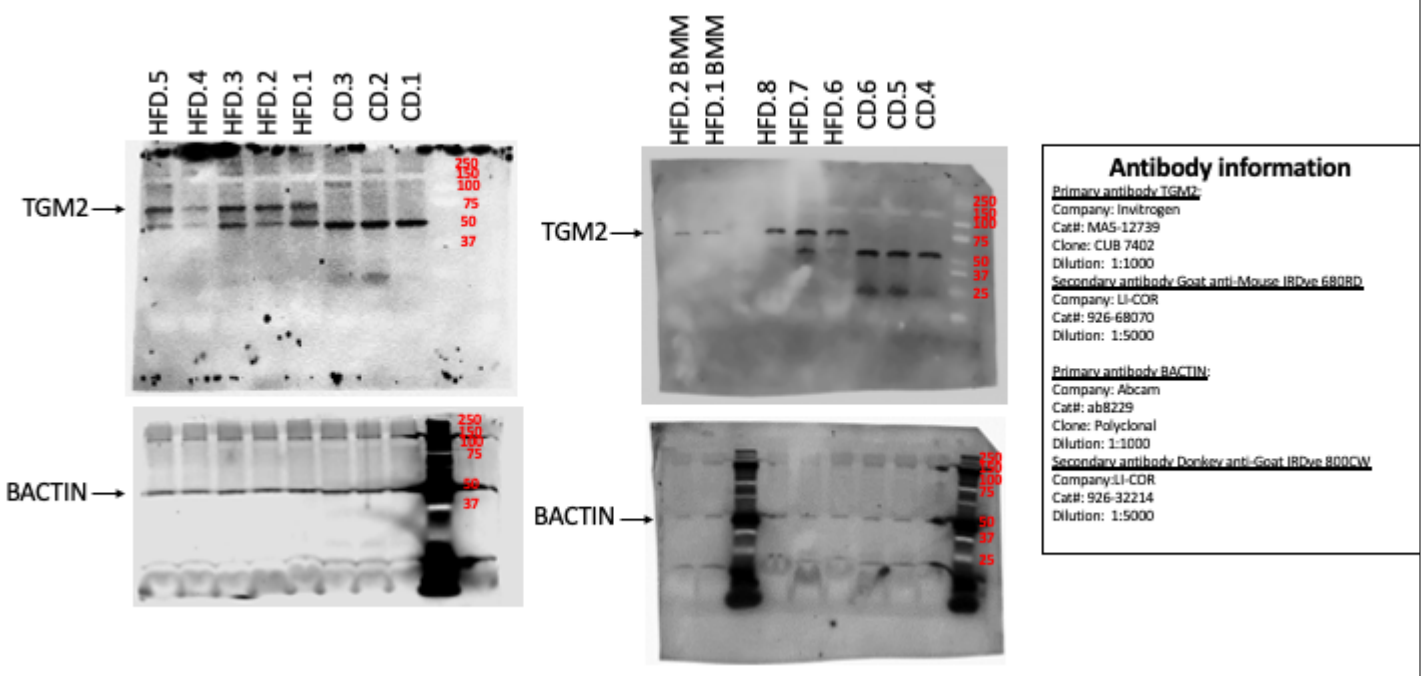

Figure 1C

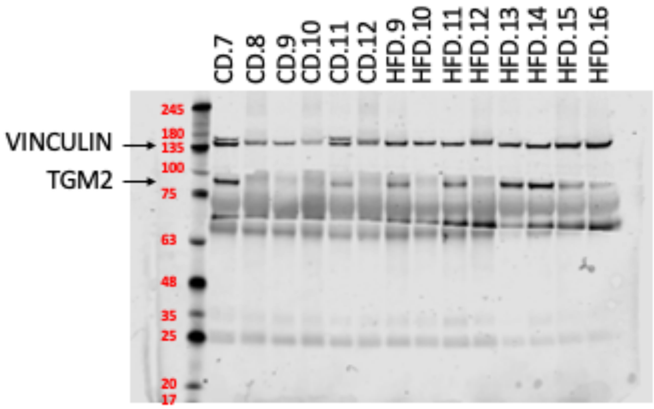

Antibody information

Primary antibody TGM2:
Company: Invitrogen
Cat#: MA5-12739
Clone: CUB 7402
Dilution: 1:500
Secondary antibody Goat anti-Mouse IRDye 680RD
Company: LI-COR
Cat#: 926-68070
Dilution: 1:5000

Primary antibody VINCULIN:
Company: Abcam
Cat#: ab129002
Clone: EPR8185
Dilution: 1:1000
Secondary antibody Goat anti-Rabbit IRDye 800LT
Company: LI-COR
Cat#: 926-32211
Dilution: 1:5000

Figure 2A

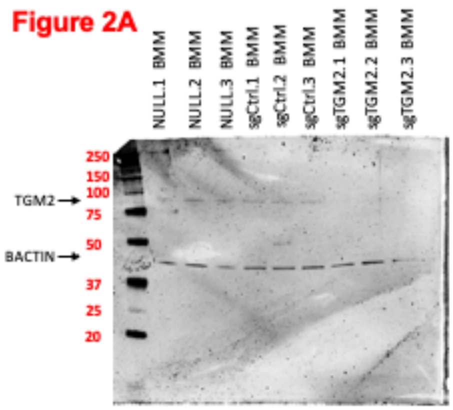

Antibody information

Primary antibody TGM2:
Company: Invitrogen
Cat#: MA5-12739
Clone: CUB 7402
Dilution: 1:1000
Secondary antibody Goat anti-Mouse IRDye 680RD
Company: LI-COR
Cat#: 926-68070
Dilution: 1:5000

Primary antibody BACTIN:
Company: Abcam
Cat#: ab8229
Clone: Polyclonal
Dilution: 1:1000
Secondary antibody Donkey anti-Goat IRDye 800CW
Company: LI-COR
Cat#: 926-32214
Dilution: 1:5000

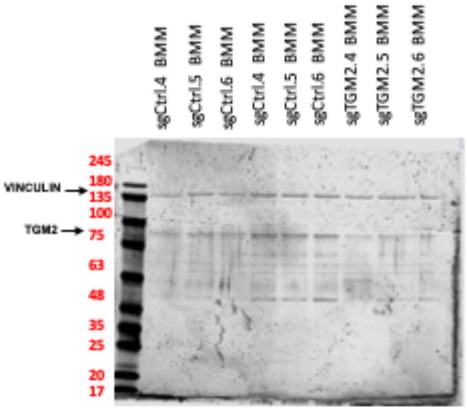

Antibody information

Primary antibody TGM2:
Company: Invitrogen
Cat#: MA5-12739
Clone: CUB 7402
Dilution: 1:1000
Secondary antibody Goat anti-Mouse IRDye 680RD
Company: LI-COR
Cat#: 926-68070
Dilution: 1:5000

Primary antibody VINCULIN:
Company: Abcam
Cat#: ab129002
Clone: EPR8185
Dilution: 1:1000
Secondary antibody Goat anti-Rabbit IRDye 680LT
Company: LI-COR
Cat#: 926-68021
Dilution: 1:5000

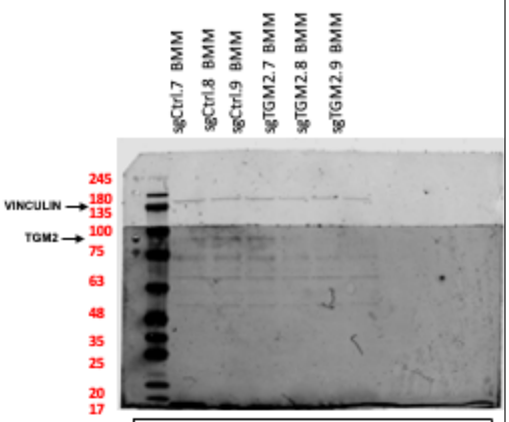

Antibody information

Primary antibody TGM2:
Company: Invitrogen
Cat#: MA5-12739
Clone: CUB 7402
Dilution: 1:1000
Secondary antibody Goat anti-Mouse IRDye 680RD
Company: LI-COR
Cat#: 926-68070
Dilution: 1:5000

Primary antibody VINCULIN:
Company: Abcam
Cat#: ab129002
Clone: EPR8185
Dilution: 1:1000
Secondary antibody Goat anti-Rabbit IRDye 680LT
Company: LI-COR
Cat#: 926-68021
Dilution: 1:5000

Supp Figure 2A

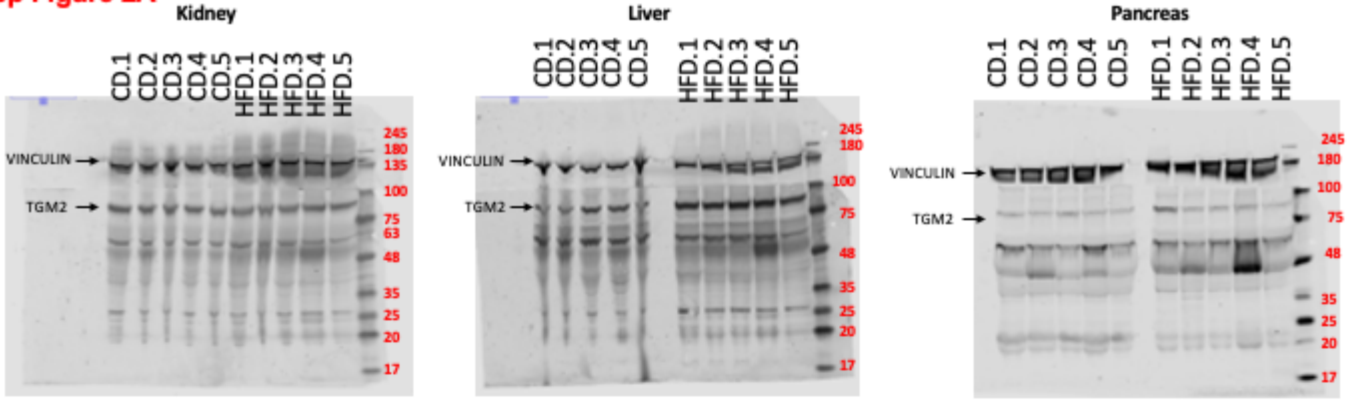

Supp Figure 2D

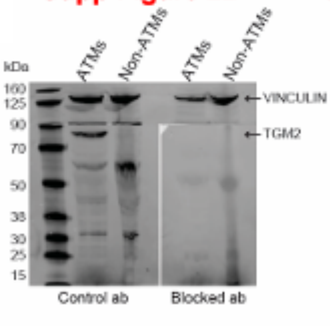

Supp Figure 2E

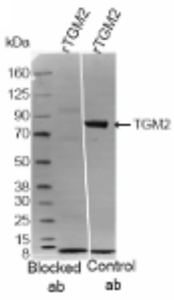

Antibody information

<u>Primary antibody TGM2:</u>
Company: Invitrogen
Cat#: MA5-12739
Clone: CUB 7402
Dilution: 1:500
<u>Secondary antibody Goat anti-Mouse IRDye 680RD</u>
Company: LI-COR
Cat#: 926-68070
Dilution: 1:5000

<u>Primary antibody VINCULIN:</u>
Company: Abcam
Cat#: ab129002
Clone: EPR8185
Dilution: 1:1000
<u>Secondary antibody Goat anti-Rabbit IRDye 800LT</u>
Company: LI-COR
Cat#: 926-32211
Dilution: 1:5000

Supp Figure 4B

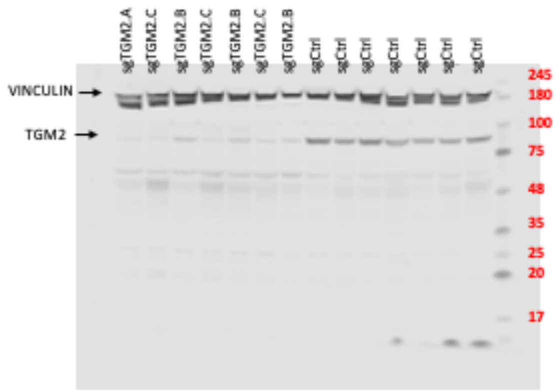

Antibody information

<u>Primary antibody TGM2:</u>
Company: Invitrogen
Cat#: MA5-12739
Clone: CUB 7402
Dilution: 1:500
<u>Secondary antibody Goat anti-Mouse IRDye 680RD</u>
Company: LI-COR
Cat#: 926-68070
Dilution: 1:5000

<u>Primary antibody VINCULIN:</u>
Company: Abcam
Cat#: ab129002
Clone: EPR8185
Dilution: 1:1000
<u>Secondary antibody Goat anti-Rabbit IRDye 800LT</u>
Company: LI-COR
Cat#: 926-32211
Dilution: 1:5000
