# Supplementary material for: Transglutaminase 2-expressing macrophages modulate adipose tissue inflammation
Source: Commun Biol. 2025 Jun 4;8:859. doi: 10.1038/s42003-025-08199-1 (PMC12137700; doi:10.1038/s42003-025-08199-1)
Supplement: Supplementary file 3 — Reporting summary [file 42003_2025_8199_MOESM3_ESM.pdf]

nature portfolio

Corresponding author(s): Jack A. Yanovski

Last updated by author(s): Apr 18, 2025

Reporting Summary

Nature Portfolio wishes to improve the reproducibility of the work that we publish. This form provides structure for consistency and transparency in reporting. For further information on Nature Portfolio policies, see our Editorial Policies and the Editorial Policy Checklist.

Statistics

For all statistical analyses, confirm that the following items are present in the figure legend, table legend, main text, or Methods section.

n/a	Confirmed	
☐	☒	The exact sample size (*n*) for each experimental group/condition, given as a discrete number and unit of measurement
☐	☒	A statement on whether measurements were taken from distinct samples or whether the same sample was measured repeatedly
☐	☒	The statistical test(s) used AND whether they are one- or two-sided<br>*Only common tests should be described solely by name; describe more complex techniques in the Methods section.*
☒	☐	A description of all covariates tested
☒	☐	A description of any assumptions or corrections, such as tests of normality and adjustment for multiple comparisons
☐	☒	A full description of the statistical parameters including central tendency (e.g. means) or other basic estimates (e.g. regression coefficient) AND variation (e.g. standard deviation) or associated estimates of uncertainty (e.g. confidence intervals)
☐	☒	For null hypothesis testing, the test statistic (e.g. *F*, *t*, *r*) with confidence intervals, effect sizes, degrees of freedom and *P* value noted<br>*Give P values as exact values whenever suitable.*
☒	☐	For Bayesian analysis, information on the choice of priors and Markov chain Monte Carlo settings
☒	☐	For hierarchical and complex designs, identification of the appropriate level for tests and full reporting of outcomes
☒	☐	Estimates of effect sizes (e.g. Cohen's *d*, Pearson's *r*), indicating how they were calculated

Our web collection on statistics for biologists contains articles on many of the points above.

Software and code

Policy information about availability of computer code

Data collection	1- ViiA™ 7 Real-Time PCR System<br>2- BMG LABTECH's FLUOstar Omega multidetection microplate reader<br>3- ODYSSEY CLx imaging System<br>4- Keyance All-in-One Fluorescence Microscope<br>5- BD LSR Fortessa Cell Analyzer<br>6- Faxitron Bioptics
Data analysis	1- QuantStudio Real?Time PCR Software v1.6.1<br>2- MARS data analysis sofware<br>3- Empiria Studio® Software<br>4- Image J sofware (NIH, Bethesda, MD): Images data analysis<br>5- FACSDiva software (v8.0)<br>6- FlowJo software (v10.8.1)<br>7- Faxitron Bioptics LLC- Vision DXA Version 2.4<br>8- We downloaded processed Seurat objects from Single Cell Portal accession ID SCP1179 generated by Sarvari et al 2021

For manuscripts utilizing custom algorithms or software that are central to the research but not yet described in published literature, software must be made available to editors and reviewers. We strongly encourage code deposition in a community repository (e.g. GitHub). See the Nature Portfolio guidelines for submitting code & software for further information.

Data

Policy information about availability of data

All manuscripts must include a data availability statement. This statement should provide the following information, where applicable:

- Accession codes, unique identifiers, or web links for publicly available datasets
- A description of any restrictions on data availability
- For clinical datasets or third party data, please ensure that the statement adheres to our policy

The datasets generated/analyzed for this study can be found in the Mendeley Data repository:
Elizondo, Diana; Patel, Tushar P; Yanovski, Jack (2025), "Data for Elizondo et al, Communications Biology submission. Transglutaminase 2-expressing macrophages modulate adipose tissue inflammation.", Mendeley Data, V1, doi: 10.17632/h54mzkg9b7.1

Research involving human participants, their data, or biological material

Policy information about studies with human participants or human data. See also policy information about sex, gender (identity/presentation), and sexual orientation and race, ethnicity and racism.

Reporting on sex and gender	N/A
Reporting on race, ethnicity, or other socially relevant groupings	N/A
Population characteristics	N/A
Recruitment	N/A
Ethics oversight	N/A

Note that full information on the approval of the study protocol must also be provided in the manuscript.

Field-specific reporting

Please select the one below that is the best fit for your research. If you are not sure, read the appropriate sections before making your selection.

☒ Life sciences ☐ Behavioural & social sciences ☐ Ecological, evolutionary & environmental sciences

For a reference copy of the document with all sections, see nature.com/documents/nr-reporting-summary-flat.pdf

Life sciences study design

All studies must disclose on these points even when the disclosure is negative.

Sample size	Sample size raging from 5-10 were implemented throughout studies to achieve sufficient statistical power according to our preliminary data, where more than 50% difference was observed. Data was analyzed using unpaired Student t-test, One-Way ANOVA or repeated measure Two-way ANOVA post-hoc were used for normally distributed data. Mann-Whitney or Kruskal-Wallis tests were employed for data not normally distributed.
Data exclusions	No data was excluded from analysis.
Replication	Total computed samples emanated from at least 3 independent experiments, which showed successful replication of the studies.
Randomization	Animals were randomly selected for diet and lentivirus injection treatments.
Blinding	Samples are ranged in numerical order along experimentation without disclosing group identity until end of analyses.<br>Flow cytometric data was collected following 96-well plate format (i.e. well 1A-H12) and analyzed accordingly without arranging samples into respective groups until end of analysis.

Reporting for specific materials, systems and methods

We require information from authors about some types of materials, experimental systems and methods used in many studies. Here, indicate whether each material, system or method listed is relevant to your study. If you are not sure if a list item applies to your research, read the appropriate section before selecting a response.

Materials & experimental systems

n/a	Involved in the study
☐	☒ Antibodies
☒	☐ Eukaryotic cell lines
☒	☐ Palaeontology and archaeology
☐	☒ Animals and other organisms
☒	☐ Clinical data
☒	☐ Dual use research of concern
☒	☐ Plants

Methods

n/a	Involved in the study
☒	☐ ChIP-seq
☐	☒ Flow cytometry
☒	☐ MRI-based neuroimaging

Antibodies

Antibodies used

Mouse IgG1 kappa Isotype Control (eBioscience; Cat#14-4714-82)
Purified Rat Anti-Mouse CD16/CD32 (Mouse BD Fc Block™) (BD Biosciences; Cat#553141)
Goat anti-Mouse, Alexa Fluor 647 (Thermo Fisher; Cat#A-21238)
Donkey anti-rabbit, Alexa Fluor 647 (Biolegend; Cat#406414)
Goat anti-Mouse, Alexa Fluor 488 (Thermo Fisher; Cat#A-11001)
Anti-Mouse, APC.Cy7 (Biolegend; Cat#405715)
Goat anti-Mouse IgG, DyLight™ 350 (ThermoFisher; Cat#62271)
Goat anti-Mouse IRDye 680RD (LI-COR; 926-68070)
TGM2 (Thermo Fisher; Cat#MA5-12739)
TGM2 (Abcam; Cat#ab109121)
B-ACTIN (Abcam; Cat#ab8229)
VINCULIN (Abcam; Cat#ab129002)
ADRP/Perilipin 2 (Thermo Fisher; Cat# CL59415294100UL)
F4/80-Alexa 594 (Biolegend; Cat#123140)
CD206 – BV605 (Biolegend; Cat#C068C2)
CD206- Alexa488 (Biolegend; Cat# 141709)
MHC Class II - BV510 (Biolegend; Cat#107635)
MHC Class II – Brilliant Violet 650 (Biolegend; Cat#107641)
IL-10 – BV421 (Biolegend; Cat#505022)
IL-10 – APC Cy.7 (Biolegend; Cat#5050335)
MHC Class II-PerCp-eFluor710 (Thermo Fisher; Cat#46-5321-82)
F4/80-PE Cy.7 (Biolegend; Cat#123113)
CD11c – Alexa594 (Biolegend; Cat#117346)
CD11c-Brilliant Violet 421 Biolegend Cat#117329
CD11b-PerCP/Cyanine5.5 (Biolegend; Cat#101227)
CD206 – Brilliant Violet 421 (Biolegend; Cat#141717)
CD4-FITC (Thermo Fisher; Cat# MA5-46784)
TCRb – PE Cy.7 (Biolegend; Cat#109222)
CD25 – PE Cy.5 (Biolegend; Cat#102010)
IFNg – Dazzle 594 (Biolegend; Cat#505846)
CD45 – Alexa Fluor 660 (Thermo Fisher; Cat# 606-0451-82)

Validation

All antibodies used were purchased from Thermo Fisher, Biolegend or Abcam and the were chosen based on manufacturers validation via testing in KO cells, or on prior published works.

Animals and other research organisms

Policy information about studies involving animals; ARRIVE guidelines recommended for reporting animal research, and Sex and Gender in Research

Laboratory animals

C57BL/6 and Tgm2tm1Rmgr/J 4-6 weeks mice were used at the beginning of the experiments.

Wild animals

N/A

Reporting on sex

Male or female mice were used for experiments.

Field-collected samples

N/A

Ethics oversight

Animal studies followed the standards of humane animal care under protocols approved by the NICHD Animal Care and Use Committee (Animal Study Protocol #21.054 and #24.054).

Note that full information on the approval of the study protocol must also be provided in the manuscript.

Plants

Seed stocks

N/A

Novel plant genotypes

N/A

Authentication

N/A

Flow Cytometry

Plots

Confirm that:

☒ The axis labels state the marker and fluorochrome used (e.g. CD4-FITC).

☒ The axis scales are clearly visible. Include numbers along axes only for bottom left plot of group (a 'group' is an analysis of identical markers).

☒ All plots are contour plots with outliers or pseudocolor plots.

☒ A numerical value for number of cells or percentage (with statistics) is provided.

Methodology

Sample preparation

SVF was harvested from eWAT, as described by Cho KW et.al., 2015 and utilized for: 1) staining of F4/80+TGM2 ATMs, 2) co-culture studies or 3) AT cell sorting. For staining approach of SVF, co-cultured or rTGM2-treated SVF, cells were harvested and incubated in Fc block Anti-Mouse CD16/CD32 (BD Biosciences; Cat#553141) 1:50 for 5 min on ice and washed 1x in FACS. Next, cells were incubated in primary conjugated antibodies diluted to 1:100 in FACS, unless otherwise specified for 20min at 4°C, as follows: MHC Class II-PerCp-eFluor710 (Thermo Fisher; Cat#46-5321-82), F4/80-PE Cy.7 (Biolegend; Cat#123113), CD206-Brilliant Violet 421 (Biolegend; Cat#141717), CD11c-Alexa Fluor 594 (Biolegend; Cat#117346), TCRβ-PE Cy.7 (Biolegend; Cat#109222), CD45- Alexa Fluor 660 (Thermo Fisher; Cat#606-0451-82), CD4-Alexa 488 (Biolegend; Cat#100425), CD25-PE Cy.5 (Biolegend; Cat#102010), IFNγ-PE/Dazzle (Biolegend; Cat#505846), IL-10- APC Cy.7 (Biolegend; Cat#5050335), IL-10- BV421 (Biolegend; Cat#505022), TCRβ- PercP Cy5.5 (Biolegend; Cat#109227), IFNγ-BV421 (Biolegend; Cat#505022).

For staining of eWAT-derived SVF ATMs sorting: Panel1 (Used for protein lysate): Propidium Iodide (Thermo Fisher; Cat# P1304MP), CD45- Alexa Fluor 660 (Thermo Fisher; Cat#606-0451-82), MHC Class II – Brilliant Violet 650 (Biolegend; Cat#107641), F4/80-PE/Cy.7 (Biolegend; Cat#123113), CD11b-PerCP/Cyanine5.5 (Biolegend; Cat#101227), CD11c-Brilliant Violet 421 (Biolegend; Cat#117329). Cells were collected in PBS and further lysed in Nonidet P-40 lysis buffer with protease inhibitor cocktail, EDTA for 1h on ice before high-speed centrifugation; supernatant was collected as protein lysate. Panel 2 (Used for RNA isolation): Live/Dead- V450 (eBioscience; Cat#65-0863-14), CD45-AFF660 (Fisher scientific; Cat#606-0451-82), MHC Class II- BV510 (Biolegend; Cat#107641), F4/80-PE (Biolegend; Cat#111603), CD14-APCCy.7 (Biolegend; Cat#123317), TCRb-PECy.7 (Biolegend; Cat#109222), CD31-PerCPCy5.5 (Biolegend; Cat#160206), CD140a-BV605 (Biolegend; Cat#135916); cells were sorted directly into TRIzol reagent for subsequent RNA isolation.

For staining of bone marrow macrophages: CD206-BV605 (Biolegend; Cat# C068C2), IL-10-APC Cy.7 (Biolegend; Cat#5050335), CD64-FITC (Thermo Fisher; Cat#MA5-46784), MHC Class II-Brilliant Violet 510 (Biolegend; Cat#107641), F4/80- Alexa Fluor 594 (Biolegend; Cat#123140). Cells were then washed in 100ul FACS 3x and re-suspended in 100uL 4%PFA for 30min on ice. Next, cells were permeabilized with 0.2% saponin and further resuspended in primary unconjugated TGM2 antibody (Thermo Fisher; Cat# MA5-12739) 1:500 at 2ug/mL or Mouse IgG1 kappa isotype control (eBioscience; Cat#14-4714-82) 1:500 at 2ug/mL overnight at 4C. Cells were then washed in 0.2% saponin 3x and incubated for 30min on ice with secondary antibody targeting TGM2 or isotype control, Goat anti-Mouse, Alexa Fluor 488 (Thermo Fisher; Cat# A-21238) diluted to 1:5000 in 0.2% saponin. Cells were washed and re-suspended in FACS buffer for data acquisition. Flow cytometric data were analyzed using FlowJo software (v10.8.1) and represented as cells% and/or cells/g, as described. Mouse IgG1 kappa Isotype Control was used to determine TGM2 staining gating strategy.

Instrument

BD LSRFortessa Cell Analyzer

Software

Data was acquired using BD FACSDiva Software (v8.0) and analyzed using FlowJo software (v10.8.1)

Cell population abundance

Adipose tissue macrophages were sorted based on Propidium Iodide-CD45+MHC Class II+F4/80+ markers' expression. Sorted cells were then protein or RNA lysed and utilized for western blot or qPCR analyses, respectively.

Gating strategy

Cell populations were gated based on the following markers:
Bone marrow macrophages: SSC+FSC+F4/80+
Polarized bone marrow macrophages: Singlets+ and then looked at single marker expression by histogram plotting
Co-cultured SVF myeloid cells: Singlets+MHC Class II+F4/80+CD206+CD11c- Or Singlets+MHC Class II+F4/80+CD206+CD11c+

Or Singlets+MHC Class II+F4/80+CD206-CD11c+
Co-cultured or rTGM2-treated SVF T cells: Singlets+CD45+TCRb+CD4+CD25+IFNg+ Or Singlets+CD45+TCRb+CD4+CD25+IL-10+
eWAT SVF sorted Adipose Tissue Macrophages: Singlets+F4/80+Live cells+CD45+F4/80+ (ATMs) or Singlets+F4/80+Live cells+CD45+F4/80- (Non ATMs)
eWAT SVF sorted cell populations: Live+Singlets+CD45+TCrb+ (T cells); Live+Singlets+CD31+CD45-CD31+Pdgfra-(Endothelial cells); Live+Singlets+CD31+CD45-Pdgfra+(Preadipocytes); Live+Singlets+CD31-CD45+tcrB-F4/80-MHC Class II+Pdgfra-CD14-(Dendritic cells); Live+Singlets+CD31-CD45+TCRb-F4/80+MHC Class II+Pdgfra-(Macrophages) and Live+Singlets+CD31-CD45+TCRb-F4/80-MHC Class II+CD14+(Monocytes)

☒ Tick this box to confirm that a figure exemplifying the gating strategy is provided in the Supplementary Information.
